# The qualitative experience of telehealth access and clinical encounters in Australian healthcare during COVID-19: implications for policy

**DOI:** 10.1186/s12961-021-00812-z

**Published:** 2022-01-15

**Authors:** Jennifer White, Julie Byles, Tom Walley

**Affiliations:** 1 Centre for Women’s Health Research, College of Health, Medicine and Wellbeing, University of Newcastle, Locked Bag 1000, New Lambton, NSW 2305 Australia; 2 Hunter Medical Research Institute, Newcastle, NSW Australia

**Keywords:** Qualitative, Telehealth, COVID-19

## Abstract

**Background:**

Adaptive models of healthcare delivery, such as telehealth consultations, have rapidly been adopted to ensure ongoing delivery of essential healthcare services during the COVID-19 pandemic. However, there remain gaps in our understanding of how clinicians have adapted to telehealth. This study aims to explore the telehealth experiences of specialists, based at a tertiary hospital in the Hunter Region, and general practitioners (GP), including barriers, enablers and opportunities.

**Methods:**

An interpretative qualitative study involving in-depth interviews explored the telehealth experiences of specialists, based at a tertiary hospital in the Hunter Region of Australia, and GPs, including barriers, enablers and opportunities. Data were analysed using an inductive thematic approach with constant comparison.

**Results:**

Individual interviews were conducted with 10 specialists and five GPs. Key themes were identified: (1) transition to telehealth has been valuable but challenging; (2) persisting telehealth process barriers need to be addressed; (3) establishing when face-to-face consults are essential; (4) changes in workload pressures and potential for double-up; (5) essential modification of work practices; and (6) exploring what is needed going forward.

**Conclusions:**

While there is a need to rationalize and optimize health access during a pandemic, we suggest that more needs to be done to improve telehealth going forward. Our results have important policy implications. Specifically, there is a need to effectively train clinicians to competently utilize and be confident using this telehealth and to educate patients on necessary skills and etiquette.

## Background

Adaptive models of telehealth (via telephone consultation, email or videoconferencing) have been rapidly adopted to ensure ongoing delivery of essential healthcare services during the COVID-19 pandemic [1]. Telehealth is the use of information and communication technology applications to provide health services, expertise and information over distance, geographic, time, and social and cultural barriers [2]. The increase in the uptake of telehealth has been unprecedented during COVID-19, and there is wide scope to consider how to integrate telehealth into routine practice going forward.

Telehealth consultations are likely to continue while we live with COVID-19 and the risk of other pandemics. Beyond these emergencies, telehealth may also have a role in providing timely care for people with multiple chronic complex conditions and people in hard-to-reach communities [3]. Changing to a telehealth delivery mode requires clinicians to navigate a different environment to attend to clinical tasks. Telehealth consults necessitate a greater emphasis on communication and skilled information exchange to effectively engage, activate and facilitate patient self-management [4]. Known challenges to the uptake of telehealth have included changes to professional roles, workflow and relationships [5, 6]. Additional barriers to telehealth include assessing a patient’s needs, technical/legal issues related to sharing protected health information, equipment limitations and integrating telehealth into existing healthcare processes [7]. Clinical concern stems from evidence that a lack of confidence in telehealth and associated technology creates a barrier to its effective use among home health nurses [8], physicians [9] and counsellors [10]. Further, the diversity of platforms and providers has led to documented inconsistencies in care [11].

Central to the context of this study is evidence of a threefold increase in the number of outpatient telehealth consultations at the John Hunter Hospital, a tertiary hospital in the Hunter Region, in March 2020 compared to the equivalent quarter in 2019. Data also highlight that, despite the Australian Department of Health recommendation that video consults be used as the primary mode of contact and that telephone should be used only when video consults are not possible [12], approximately 90% of Australian telehealth consultations in 2020 took place over the telephone [13]. In order to understand the complexity of a rapid transition to telehealth management and how clinicians have approached clinical interactions and decision-making, this study aimed to explore the telehealth experiences of specialists and general practitioners (GP). Such understanding is critical if we are to improve and sustain quality service delivery to patients using telehealth, translating telehealth into a broader system of care with the methods and tools required to support it.

## Methods

Qualitative research investigates the subjective nature of social reality and the relationships between individuals and the institutions and society in which they live [14]. This qualitative study employed the use of semi-structured interviews and was informed by the Consolidated criteria for reporting qualitative research (COREQ) checklist [15]. Hunter New England Local Health District specialists based at the John Hunter Hospital (JHH) and John Hunter Children’s Hospital (JHCH) and GPs who provided outpatient and community-based services were eligible for inclusion. Representation of the different outpatient speciality clinics at the JHH and JHCH was sought. Participants were invited to participate by response to an email distributed by their heads of department and the director of a large, local general practice, including detailed study information. All participants provided written informed consent. Recruitment occurred between August and November 2020. Approval for this project was obtained from the Hunter New England Health Human Research Ethics Committee (2020/ETH01732).

### Data collection

Semi-structured interviews (specialist *n* = 10, GPs *n* = 5) were conducted by a single interviewer (JW) using an interview schedule [16]. Interviews began by asking participants to share their “story” of transition to telehealth, and subsequent questions explored their experience of clinical interactions and decision-making using telehealth, including perceptions of benefits, barriers and opportunities (see Table 1). The semi-structured nature permitted flexibility for participants to elaborate upon or cover important topics that would not have otherwise surfaced [17]. Identified themes informed continuing data collection, and sampling continued until thematic saturation (two co-coders agreeing that no new themes were emerging) was achieved.

**Table 1:** Question guide

Question	Prompt
Can you please start by providing a brief overview of your current position and how the pandemic has impacted you?	At what stage did you introduce telehealth? What was needed to establish a telehealth service?
What has been your experience of telehealth during COVID-19?	What types of clinical encounters have you provided through telehealth? Why? Why not? Did you change anything about your consult? Any other changes other than telehealth uptake? Did telehealth work as you hoped?
What clinical interactions do you think cannot be effectively conducted through telehealth?	How did you manage these during COVID-19? Any patients postponed?
How did the telehealth consult compare to usual care?	Communication? Verification without hands-on assessment? How did you think patients received your consult? How did you manage patients with complex needs? What did you learn/change over time as you provided consults over telehealth? Any team consults?
Have you previously used telehealth?	Expand? Do you feel confident using telehealth? What would help you? How did you test patient retention and understanding, if at all? Do you think you would benefit from additional training?
Did you encounter any adverse events?	Please expand? How did you manage this?
What do you feel are benefits of telehealth?	Expand?
Do you think there is potential and value in extending telehealth beyond the COVID-19 period?	Expand? What is needed? Any complementary services required? Complex care and team?
Do you have any further comments?	

### Data analysis

Semi-structured interviews, ranging from 30 to 60 minutes, were recorded with the participant’s permission and transcribed verbatim with identifying data removed. Two authors (JW, JB), one an occupational therapist and the other a gerontologist, coded data using an inductive thematic approach [18]. At the level of initial coding, both authors read the transcripts multiple times and made notes. Transcripts were then coded line by line, describing and interpreting emerging categories and searching for differences and similarities. Following team discussion, the primary author developed a single codebook, and each code was issued with a four-letter label or code to facilitate data retrieval between the transcripts (for example, symptoms of confidence was labelled “CONF”). The next step involved examining the relationship between codes in the context of the research question in order to form themes. Consistency of findings was upheld through discussion of interpretations between researchers to confirm codes and categories. Any differences in researcher perspectives were resolved by negotiation and, if necessary, regrouped and recoded until consensus was reached. New codes were then fed back into the analysis to cross-check codes and themes and develop an overall interpretation of the data [19]. Trustworthiness of our data was achieved using several strategies, including immersion in data, reflexive analysis and peer debriefing [20, 21]. These strategies ensured that the researchers remained open to the data and did not demonstrate bias during the data analysis as a result of preconceptions inherent in the researchers’ clinical status and experience [20, 21]. Coders captured exemplary quotes supporting each theme.

## Results

Clinician demographics are outlined in Table 1. Ten specialists including geriatricians, interventional cardiologists, general physicians, neurologists, endocrinologists, paediatric specialists and five GPs participated in this study. Limited participant characteristics are given to protect anonymity.

This programme of research identified key findings across six areas:

i. Transition to telehealth has been valuable but challenging.
ii. Persisting telehealth process barriers need to be addressed.
iii. Establishing when face-to-face consults are essential.
iv. Changes in workload pressures and potential for double-up.
v. Essential modification of work practices.
vi. Exploring what is needed going forward.

### Transition to telehealth has been valuable but challenging

The experience of transition to telehealth varied among participants. As this was a tertiary referral hospital, many specialists already provided telehealth services to designated rural and remote local government areas. Likewise, GPs reported that telehealth “is something that we have always done… [we were] just never reimbursed for it. There has always been calls at the end of the day, conversations discussing results, follow-ups that aren’t face to face but are urgent. You know, there is a role for those” (Participant [P] 13, GP).

However, many participants reported feelings of “heightened anxiety” (P4) when trying to monitor and implement “constantly changing” (P10, GP) government directives in response to COVID-19. Specific challenges related to reading and responding to large amounts of communication received from the workplace and government about required work practice changes.

Right from the beginning … multiple, multiple emails and sort of communications about what should and shouldn’t happen, protocols to become up to date with, exposures to new terminology. (P2, specialist [S])

For most participants, the transition to telehealth reportedly happened “overnight” (P4, S) and with limited forewarning, which meant that essential processes and infrastructure were not in place. Participants reported a lack of access to essential equipment, including phones with a speaker option, computers with video capacity or multiple screens, and adequate outgoing phone lines. In many cases participants ended up buying and using their own equipment.

Most participants experimented with different modes of communication during the transition to telehealth. Over time, most participants opted to conduct phone consults, as videoconferencing modes were considered unreliable and required extra time to set up, and often failed, which often led to swapping to the phone.

Me and my colleagues did experiment with using some of the video formats, but …so much time was lost trying to get things to operate or work. Eventually I think everybody abandoned it [and used the phone]. (P12, GP)

Patient familiarity with different telehealth modes was perceived to vary, irrespective of age. Participants indicated that transition to successful telehealth consults was closely linked with patients’ familiarity with and access to the various modes of telecommunication using video features.

Most of our patients don’t have mobile phones or are confident managing of them…or a lot of them don’t have somebody that can help them to set it up. (P2, S)

What has astonished me is how really poor the tech-savvy [aware] people are [with telehealth] … even 19- and 20-year-olds. (P8, S)

### Persisting telehealth process barriers need to be addressed

Numerous factors were identified as barriers to engaging with telehealth. Most notable was the significant time required for staff, typically administrative staff, to schedule appointments in both hospitals and GP practices. This required knowledge of the modes of telehealth that patients had access to, as well as the correct patient contact details to enable login. Participants often spent extra time helping people access programmes and problem-solve telehealth access issues.

One of the main constraints is technology… the patient needs to be primed, and that person may or may not have the ability to prime themselves to get on [telehealth]. It takes about 10 minutes for us to get the person prepared, and 10 minutes is a long time for us to waste on, and in a busy clinic, when you have 30 patients coming, a lot of people are waiting. We are running late by an hour and a half, I then have to invest 10 minutes of my time to get this person prepared. It was very unsatisfactory re coordination aspects. (P15, S)

The initial nature of the booking system was ad hoc and “not clearly defined” (P5, S), reportedly leading to errors and confusion. Scheduling of appointments and dealing with a virtual waiting room was confusing and problematic for both participants and patients, and the absence of a system to keep patients updated regarding their appointment meant that patients were often not available at the negotiated time, especially if participants were running late or early.

Internet connection issues were common to participants and patients, which had the potential to create significant delays. Overall, patients were more likely to lack enough data or bandwidth. There was a perceived reliance on family or carers to facilitate a consult and help provide data, which was considered less than ideal and likely to create additional financial burdens. However, government directives often meant that “family members weren’t there [to help], as they would have been normally” (P1, S).

A lot of [older] people don’t have internet, so we have to access it from the son or daughter’s phone. I am not sure whether they were wanting to do that—just thinking how much data you can use in a 45-minute consultation. I am not sure whether family members would have wanted to use all their data in that way. (P1, S)

#### Telehealth procedures and etiquette

In contrast to the commitment and preparation required from patients to attend a face-to-face appointment, participants expressed frustration that patients appeared to place less importance on preparing for and attending telehealth consults, for example, by adhering to appointment times and environments that were free of distractions.

Yeah, it is such a waste of time, you know. You have opened the notes, you have read the notes, now you want to deal with the problem. If they are not there and you ring back 2 hours later, you then have to reopen the notes, refresh your mind with that patient. Yeah, that was a really big challenge, and also I just feel like people probably do not value it all that much. I do not think our patients value it all that much. (P10, GP)

Several clinics had existing or had developed processes/education to help patients set up and engage with a telehealth appointment, such as how to download programmes, who to call for support, and ensuring a quiet space/room with few distractions. However, participant reports suggested that patient responsiveness to this support, which aimed to facilitate a quality consult, was variable. Furthermore, many patients did not come prepared for their telehealth appointment by obtaining essential pathology or scans before a scheduled consult, as they would do in a face-to-face consult. This not only created significant delays and double-up when appointments needed to be rescheduled, but also placed patients at risk when areas of concern were not investigated in a timely manner.

I have had a lot of people not attending their blood tests or not attending their ultrasound scans or their surveillance colonoscopy. So, there certainly has been delay in some things. I haven't had a sinister outcome from that yet… touch wood. (P13, GP)

[Many patients have] nothing prepared for this consultation. So, I wasted all my time and effort, no decisions could be made on that day…. I am actually quite disappointed that there is not a system of coordination before a telehealth happens. Someone needs to tell them what the expectation of the doctor is. (P15, S)

Similarly, many patients “would not pick up the phone” (P11, GP) at the scheduled time, which required participants to make multiple calls. Alternatively, patients expected participants to call when it suited them.

[Patients may say] I have just come out of shower, can you wait for 5 minutes. That is completely unacceptable. I mean we are wasting our precious time. (P15, S)

Many patients also attempted to participate in a consult when distractions—which were previously minimized during a face-to-face consult—were present. Distractions were wide-ranging, such as when children were present at home or in a car, or when in a busy/noisy environment such as a shopping centre. In these scenarios, clinicians often terminated and rescheduled the appointment, especially when issues concerning confidentiality were evident.

I had a consultation with someone who was in the middle of a paddock and, you know, we have had conversations with people that were shopping … we are not going to have this conversation. This is, you know, confidential stuff. (P8, GP)

### Establishing when face-to-face consults are essential

#### Assessment accuracy

All participants preferred to see new and complex clients face to face due to the imperative for obtaining a clear history, undertaking physical examinations and establishing rapport. Participant confidence in dealing with an incomplete picture varied such that some experienced greater anxiety. Most participants arranged for a patient to come in for an in-person consult if there were any concerns; however, the waiting list was often more prolonged for specialists.

It [incomplete assessment] gave me a lower threshold to bring them in [for an in-person consult]. (P10, GP)

For some, concern stemmed from putting interim measures in place or delaying treatment until a patient could be seen in person.

You may decide not to embark on treatment straightway, to see how they went, whereas if you had seen them in person, you would likely start a treatment more immediately. (P1, S)

Participants also expressed concern that patients were minimizing their issues over telehealth consults which was often linked to them not wanting to be a burden during a challenging time.

I do think a number of people minimize their symptoms because they didn’t really want to bother me. (P6)

#### High-risk patients

Some participants expressed concern at the potential to miss aspects of an assessment, since telehealth relied on patient self-report of symptoms rather than observation.

Yes, very early on I missed a cerebellar stroke, so that was a bit unpleasant for me. You just do not pick up from those cues of seeing them in the waiting room, standing up, walking in, where you get [visual information] …. The consultation starts in the waiting room, so you are missing out on that completely [in a telehealth consult]. (P10, GP)

Observation and physical assessment were integral to a consult, including noting social aspects, unusual neurological presentations (eye movements) and movement disorders, sensation and movements that caused patients to be at risk of falls, insulin injection sites, joints (rheumatology) and allergies.

I am fearful that I may have missed things that could be important…for example, I was talking to a woman, and by the end of the consultation the penny dropped that she was morbidly obese [which impacted clinical advice]. But nowhere on the GP referral or within her conversation or conversation with her husband had that been described. (P1, S)

Face-to-face communication was also deemed more beneficial when treating people who were deaf, brain-injured or cognitively impaired. Similarly, participants noted that providing education and supporting patients in understanding and adhering to education regarding chronic disease management was also made difficult when face-to-face communication was not possible. In these clinical scenarios, participants preferred to offer an in-person consult.

If you really want to reinforce exercise, diet, alcohol, not smoking, that kind of thing, if you really want to motivate the patient to make some change, get them in. (P12, GP)

Several critical care scenarios were noted where the inability to see someone face to face had the potential for overlooking patients at risk, particularly children and the elderly.

I gauge a lot from seeing the patient’s face and seeing how they move …. I mean, it’s just simple things like looking at their skin or their feet, the condition of their feet. You can tell a lot about how somebody is being cared for ,you know… things like vulnerability [to abuse]. (P2, S)

Specifically, there was a need to assess patients at risk of abuse and to be able to create a separate time and space where the patient and family could be spoken to separately.

Concerns around the patient’s vulnerability in the setting of family disharmony, a lot of tension in the household and things. Normally I would separate the patient from the family members with my community intervention nurse separately and see them separately, and so we could sort of gauge some aspects of the consultation in private. (P2, S)

Adequately dealing with abused patients and patients with mental health issues reportedly required a close therapeutic relationship and the time/opportunity to adequately deal with any emergent distress. All participants acknowledged the difficulty in detecting patient distress and providing support through telehealth, especially telephone, and that this was a potential barrier to telehealth.

So, they often get quite distressed, and you need to be able to manage that safely, because normally if they are going to transition from the clinic room through the corridors and social environments back to the car, etc., that allows a bit of processing. But if they go to their lounge room with no transition at all … they can end up going back to crying for a long time if you haven’t helped them kind of resolve their emotion in the session. (P7, S)

Sometimes I hear silence, I don’t know this person is upset until I hear them crying. But if they come to me, I can make out very easily this person is getting upset, anxious, then we can really get into the issues that has prevented them from engaging in a proactive healthcare. It may be distress regarding the diagnosis, it may be denial about their condition, so unless we address those aspects properly, you do not actually make a clinical outcome better. (P15, S)

#### Therapeutic relationship

Developing a therapeutic relationship through telehealth was problematic and a key barrier reported by all participants, although not insurmountable. Telehealth, even with video capacity, made it difficult or delayed the ability to engage with a patient, read body language, respond to communication cues, and engage in conversation that allowed for a relationship to develop as well as elicit helpful supporting informative, for example, regarding socialization. In most scenarios, however, patients were seldom given the choice of telephone or video.

Well, a telehealth consult is really a conversation to try and get out what it is you need to know. One of the problems is you cannot read body cues—you have got to be quite concise in what you ask because it is not easy to interrupt, whereas in an interview there are other cues that will allow them to know that you are ready to ask another question or perhaps to ask something more on that subject. You cannot easily do that on phone. (P6, S)

Participants also felt that patients were more likely to provide additional, valuable information during an in-person consult, often when talking informally. Many GPs perceived that patients “feel more at ease to bring up other complaints they might not have thought were a big deal, and sometimes they are not. But other times, you know, it might be something that concerns us more than the patient. Mental health is a big one as well. There are a lot of times where people will come in face to face for a script or some other condition, and then you end up having a good chat about their mental health. I would not get that over the phone at all” (P11, GP).

### Changes in workload pressures and potential for double-up

Telehealth was reportedly a time-consuming process, and extra time was taken in the initial stages to accommodate new processes and workflow issues. For example, administrative staff needed extra time to set up appointments, problem-solve patient access issues or rebook appointments. Participants indicated that while telehealth appointments were shorter in length, it was common to spend additional time before and after a consult. For example, a significant amount of time was spent compensating for a lack of information, thus requiring time to review reports and referrals prior to a consult. Similarly, additional work in the early transition to telehealth often required participants to spend extra time, for example, writing lengthy letters to GPs to ensure adequate follow-up and to outline any assessment omissions, ordering/faxing tests and scripts, and emailing or phoning GPs or other staff with instructions or for verification.

I finish a consultation and I am definitely not as confident as I would be when I am assessing face to face, but I guess I make it clear in my communications it is a limited assessment in some respects. (P2, S)

That is a disappointing aspect of this whole coordination of telehealth that we have to ring the pharmacy, we have to ring the GP practice, we have to ring the pathology, we have to then send the scripts to this place, ring and tell them that the fax is coming. (P15, GP)

Shorter consults that were not face to face also had the potential for errors if concentration was lost during a busy clinic, especially when trying to take in information from various modalities.

It is far easier to make a mistake on telephone conversation, because the information that is coming is far less. So, I think telephone consultations are pretty hopeless. For instance, once I got the medications wrong. I must have been concentrating on something else. And they are much quicker… telephone conversations are like a quarter of the time. (P3, S)

We might also make mistakes. I have seen that sometimes you call the wrong person and you go through the consultation and then you realize you are in the wrong folder. (P15, S)

Overall, many felt that telehealth resulted in a doubling up of their time and efforts.

I need to see this guy, it has been two appointments I have seen him on a telephone basis. I really do not know what is happening. I need to bring him back and see him. It does create extra work. (P5, S)

### Essential modification of work practices

All clinicians readily modified their work practice and communication style through trial and error in response to adopting a telehealth practice. For some this meant modifying standardized assessments for use over the phone such as abbreviating the Mini-Mental State Exam (MMSE) cognitive assessment to obtain some semblance of a cognitive screen, because “there aren’t many validated tools for cognitive assessment over the phone” (P2, S). Other strategies reportedly included asking more detailed questions in order to elicit correct information and compensate for a lack of visual cues or relying on family and carers for clarification.

I ask them specific questions: what happened about your blood sugars, what happened with your blood tests. I am looking for answers. We need to have clear questions, as not all patients have clear questions to give clear answers. We should try to explore their questions and their concerns. (P5, S)

One clinician with extensive experience with telehealth reportedly increased the number of pauses to allow a patient to speak, since the nature of telehealth often meant that people spoke overtop each other.

[Face to face] I can pick up on subtleties in body language and I can see or hear [a patient trying to speak] and I know now it is my turn to stop talking. … I stop talking a lot earlier [during telehealth] than I would normally stop talking and check if you have got anything that you want to say. (P8, S)

Participants spent additional time seeking verification from patients such as encouraging patients to write things down or asking them to repeat what had been said.

When I think I am not sure if my patient is able to understand exactly what I am saying, then I ask them to please, get a pen and paper, and write this down. (P4, S)

### Exploring what is needed going forward

All clinicians reported that telehealth was inferior to face-to-face consults but had a role going forward. Telehealth was considered most appropriate for patients where there was an existing therapeutic relationship, and for “follow-up things or for things where it is a very easy diagnosis, you know” (P8, S). Participants greatly appreciated being remunerated for time spent on the phone providing follow-up, confirming an ongoing treatment plan or writing scripts. Likewise, there was a perceived benefit for an initial consult where blood work, scans, and so on were requested prior to further assessments or prior to a well-defined procedure. These had a mutual benefit for patients in not requiring them to travel, wait for long periods and pay for parking. Specific benefits were noted for children at risk of missing a day of school at a time, significantly disabled patients where transport was difficult, and older people who had the potential to experience fatigue and confusion.

We are trying to keep kids in school, so if they have to have a day off to travel, that’s a problem. If we can telehealth to a computer at school, then that is better. (P8, S)

Some participants felt that efficiency could be improved by triaging patients ahead of time. In this way it could be determined whether it was essential that they attend in person, to ensure that care was maintained and not delayed.

I know this month I have been triaging patients, we get about 30 to 40 referrals a week in diabetes. I can easily say which one needs to come definitely and which one does not need to come. We can triage it and then that is not the problem. (P15, S)

However, participants emphasized that if increased telehealth services were expected, then more infrastructure was needed regarding equipment and staffing.

We are just expected to absorb it. That is the difficult part if you expand telehealth because telehealth can go anywhere, but at this end somebody needs to enhance us, and we have not had any enhancement. (P15, S)

Telehealth also provided the scope to provide continuity of care by streamlining services in facilities for the aged, supporting palliative care and other complex care and social issues, such as busy mums.

I think the mum was anxious, there was nothing wrong with the baby. But for her it was really good doing a video call, just reassure. Reassure is part of follow-up anyway. (P10, GP)

Participants noted the potential benefit in establishing telehealth clinics separate from in-person clinics in order reduce the demand experienced by each clinic. For example, some participants reported feeling greater cognitive fatigue with telehealth consults, due to the need to anticipate problems, check and double-check information sources or monitor different screens (such as electronic records and video).

It is the constancy of, you know, the next time that you contact someone, are they going to be there, what technical issues …I am trying to describe something to someone, you know, or have a lot of images to show them. [When they are face to face] it is easy to show them images. (P8, S)

## Discussion

As with other countries, telehealth has enabled Australians to stay connected with the healthcare system during COVID-19. Participant reports highlighted that the use of telehealth has increased dramatically. Consistent with international reports, convenience benefits were noted in maintaining health access for patients and reducing the burden of travel and waiting times [22]. Likewise, participants were reimbursed for essential work they were already conducting over the phone [23]. Echoing previous research, this study identified limitations of telehealth which resulted in the delivery of care where participants experienced challenges with booking processes and internet connection, and poor patient computer literacy [24, 25]. These may be magnified in patients from low socioeconomic and culturally and linguistically diverse backgrounds [26, 27]. Overall, participants were required to compensate for a deficient clinical history, struggled to establish a therapeutic relationship, and spent extra time post-consult to follow up on referrals and scripts.

Communication barriers due to the lack of nonverbal communication and information exchange in telehealth have been well established [28]. Evidence shows that up to 55% of the impact of consultation is attributed to visual and nonverbal communication [29]. In addition, evidence shows that patients frequently misunderstand health information given by phone [30]. Poor health literacy is exacerbated in people with mental health issues [31] and sensory conditions such as altered vision and hearing [32]. A key finding of this study was that participants self-initiated modification of work practice to ameliorate for potential communication deficits, such as more detailed questioning and seeking verification. However, clinicians accepted that clinical care given to patients using telehealth was different, and often delayed, in comparison to care given face to face and that it was difficult to establish a therapeutic relationship. Therapeutic relationships are complex and subjective, requiring patient and therapist collaboration to optimize outcomes, and previous research highlights the need for training [33, 34]. Furthermore, while telehealth competencies exist in the provision of telehealth across various professional groups [35], few clinicians are formally trained to deliver their professional services using telehealth [36]. Consistent with growing evidence, we posit that the effective use of telehealth requires appropriate education of patients and clinicians about the safe and effective use of telehealth for the provision of high-quality patient-centred healthcare [37–39].

Previous research has demonstrated that patients are often satisfied with telehealth, the quality of care and convenience [40, 41]. A recent systematic review identified barriers and facilitators to telemedicine [42], with key barriers including access to and quality of technology, lack of seriousness, difficulty expressing difficult emotions, poor body language, and communication and scheduling conflicts [42]. However, results from our study suggest there was a perceived lack of respect for and preparation before a telehealth consult among patients, which led to unnecessary duplication of consults and delayed assessment. Indeed, previous research suggests that people from rural areas, those with low literacy or low socioeconomic status, or those from culturally and linguistically diverse backgrounds find telehealth the least accessible and may benefit from education [43].

All participants acknowledged that a telehealth appointment was better than no appointment at all. However, going forward, telehealth services were deemed to require improved software which provided booking efficiency, data security and data transfer. With greater expectations of seeing people who lived in closer proximity came the need for more staffing and infrastructure to support this. As supported by a recent systematic review of the role of telehealth during COVID-19, improved administrative processes, such as having a triage system, would allow for more accurate identification of people who needed to be seen person [44].

## Strengths and limitations

While our approach provided the opportunity to obtain diverse perspectives on what current and future telehealth services should look like, the use of purposive sample may have resulted in an invested sample. Likewise, our sample was small and selected from a single urban location. We also did not capture the patient perspective. Future research should explore the patient perspective, including people from culturally and linguistically diverse populations. In addition, research should be prioritized to ensure the consistent quality of telehealth consultation.

## Conclusion

With the unprecedented increase in the uptake of telehealth, there is an opportunity to integrate telehealth into routine practice, potentially improving inequities and inefficiencies in the delivery of care for patients. This study highlights barriers to establishing a therapeutic relationship using telehealth, which was compounded by the lack of visual and nonverbal communication, internet connectivity and computer literacy. A further challenge was a perceived lack of respect for and preparation before a consult by patients, which led to further delays in the provision of timely care, such as the need to rebook appointments.

Our results suggest several policy areas for attention to support telehealth, including further investment in information technology infrastructure across health services administrative support to facilitate changes in practice and workflow. Effective communication is paramount to delivering patient-centred care, and cultivating this skill is a vital component of telehealth. Given that the use of telehealth, both phone and video, is increasing and is likely to continue while we live with COVID-19 and the risk of other pandemics, we would point to the importance of promoting communication competency through training of health professionals and patients in the use of telehealth.

## Data Availability

The qualitative data used and/or analysed during the current study are available from the corresponding author on reasonable request.
